# Recent progress in satellite cell/myoblast engraftment – relevance for therapy

**DOI:** 10.1111/febs.12273

**Published:** 2013-04-24

**Authors:** Deborah Briggs, Jennifer E Morgan

**Affiliations:** The Dubowitz Neuromuscular Centre, UCL Institute of Child HealthLondon, UK

**Keywords:** cell therapy, Duchenne muscular dystrophy, mdx mouse, muscular dystrophy, myoblasts, satellite cells, skeletal muscle regeneration

## Abstract

There is currently no cure for muscular dystrophies, although several promising strategies are in basic and clinical research. One such strategy is cell transplantation with satellite cells (or their myoblast progeny) to repair damaged muscle and provide dystrophin protein with the aim of preventing subsequent myofibre degeneration and repopulating the stem cell niche for future use. The present review aims to cover recent advances in satellite cell/myoblast therapy and to discuss the challenges that remain for it to become a realistic therapy.

## Introduction

Muscular dystrophies comprise a large group of heterogeneous genetic disorders characterized by progressive muscle weakness and degeneration, which vary with respect to severity, the muscle groups affected and the involvement of the heart 1. Duchenne muscular dystrophy (DMD), the most severe form, is caused by mutations in the gene for DMD, leading to a near absence of functional dystrophin protein 2,3. Dystrophin is located beneath the sarcolemma; it functions to assemble the dystroglycan complex at the sarcolemma and to connect the internal cytoplasmic actin filament network and extracellular matrix, thus providing physical strength to the muscle fibre 4. Myofibres lacking dystrophin are easily damaged, leading to satellite cell-mediated repair. However, the repaired/regenerated myofibres in turn degenerate, leading to chronic muscle degeneration and regeneration, as well as exhaustion of the satellite cell pool. This results in the eventual loss of muscle fibres and their replacement by fibrotic and fatty tissue, compromising normal muscle function 5.

Satellite cells are the principal skeletal muscle stem cell. They reside between the sarcolemma and basal lamina of muscle fibres and are mitotically quiescent until required for growth or repair. Upon receiving activation signals, they rapidly proliferate to produce a pool of myoblasts that fuse with each other to form nascent myofibres and/or with damaged fibres to repair them. A small minority do not differentiate but, instead, re-enter quiescence to maintain the stem cell pool 6. Satellite cells are extremely efficient at repairing muscle; several thousand myonuclei can be generated from a small number of transplanted satellite cells contained on a single fibre 7 and even from just a single satellite cell obtained by fluorescence activated cell sorting 8. Transplanted satellite cells can occupy the satellite cell niche and participate in future rounds of regeneration, indicating self-renewal and confirming their stem cell status 7.

There is a body of experimental evidence to support the hypothesis that not all satellite cells are functionally equivalent. Only a minority of satellite cells contributes to muscle regeneration 7,8 and recent data from satellite cell transplantation experiments have suggested that there are two populations of satellite cells. One population is responsible for myonuclei addition during growth and general muscle maintenance throughout life; these satellite cells are present in greater numbers in growing muscle, are diminished with age, and are more numerous in adult males compared to females. The second population is formed by those satellite cells that are activated by severe muscle injury and survive transplantation; they are present in similar numbers from birth to old age and do not differ between male and female mice 9. A subpopulation of satellite cells has been shown to produce distinct daughter cell fates by asymmetrically segregating template and newly-synthesized DNA strands 10; these may correspond to the ‘stem’ satellite cells that are capable of contributing to muscle regeneration and functionally reconstituting the satellite cell compartment 7.

The findings that stem cells other than satellite cells (derived from muscle, bone marrow, the interstitum or the circulation) could also contribute to muscle regeneration led to studies moving away from using satellite cells/myoblasts, towards atypical stem cells 11,12. Of the cells investigated, those with the greatest potential appear to be mesoangioblasts 13,14, pericytes 15,16 and CD133+ cells 17–19, as a result of their ability to migrate through the vasculature (a major limitation of satellite cell/myoblast therapy), to contribute to considerable muscle regeneration and to engraft into the satellite cell niche. Indeed, mesoangioblasts are currently being tested in a clinical trial for DMD, under the direction of Guilo Cossu (Division of Regenerative Medicine, San Raffaele Scientific Institute of Milan, Italy). Other recently described but less well characterized cells, which may also hold some promise, are PW1+ cells and amniotic fluid stem cells. PW1+ muscle resident interstitial cells reportedly have a regenerative capacity similar to satellite cells and can reconstitute the satellite cell niche; however, so far, they have only been isolated from mouse muscles and injected intramuscularly 20. Amniotic fluid stem cells 21 are multipotent cells capable of undergoing myogenesis and proof-of-concept studies have shown that they make some contribution to muscle regeneration in mouse models after local or systemic delivery 22,23. The recent discovery that, in the adult mouse at least, Pax7+ satellite cells are the only cells that can regenerate skeletal muscle (i.e. their conditional genetic ablation completely prevents regeneration 24–27) suggests that the myogenic contribution of other stem cells is either negligible or requires paracrine factors released by satellite cells for them to enter the myogenic programme, or that ablation experiments result in excessive disruption of muscle tissue, in turn perturbing the homeostasis of other stem cells. This may help explain the apparent discordant findings of Dellavalle *et al*. 16, who elegantly demonstrated the fusion of muscle resident pericytes with developing myofibres, as well as pericytes, entering the satellite cell compartment during postnatal growth. The re-establishment of the satellite cell as the principal endogenous muscle stem cell comes at a time when much effort is focused on cellular therapies. Recent advances in overcoming the limitations of myoblasts, with the aim of improving their regenerative capacity, are the focus of the present review.

## Myoblast cell therapy

### Failure of early myoblast transplantation clinical trials

Cell therapy (i.e. the delivery of myogenic cells to enact muscle repair) has been considered as a potential therapy for DMD for many years, ever since Partridge *et al*. 28 demonstrated that donor myoblasts could fuse with host myoblasts, suggesting the possibility of functional restoration in defective fibres. The pivotal discovery that donor heterologous myoblasts could restore dystrophin expression in the dystrophin deficient mdx mouse 29 set the precedent for a number of human clinical trials in DMD patients in the 1990s 30. Disappointingly, little or no dystrophin restoration was observed in the injected muscles and no functional improvements were discerned 31–38. The failure of the trials was subsequently attributed to several factors, including the rapid cell death of the majority of cells within a few hours of transplantation, the limited migratory capacity of transplanted cells and a lack of immune suppression leading to graft rejection 12. It is also now known that myoblasts are not as efficient as their parent satellite cells. Standard culture greatly reduces their regenerative and self-renewal capacity 39.

### Overcoming problems

Strategies to overcome some of these problems include improved immunosuppression, the injection of more cells but in smaller volumes to prevent ischaemic necrosis, and high-density injection protocols to aid migration 40. Using these improvements, a recent phase I clinical trial for DMD delivered a large number of allogeneic myoblasts using multiple injections (high-density injection protocol) to the biceps brachii, under continuous immunosuppression by tacrolimus (FK506), to avoid rejection. Long-term expression of donor-derived dystrophin was detected in 27.5% of fibres 1 month after injection and, in 34.5% of fibres, after 18 months 41,42. Although promising, this was only achieved in one patient, repair was localized to the injection sites, long-term immunosuppression is required and the protocol is only applicable to easily accessible small muscle groups 43.

Clinical trials, using autologous myoblasts, for muscular dystrophies that affect only subsets of muscles, namely oculopharnygeal muscular dystrophy (ClinicalTrial.gov identifier: NCT00773227) and facioscapulohumeral muscular dystrophy (under the direction of C. Desnuelle, Centre Hospitalier Universitaire de Nice, France) are currently underway (initiated in 2005). These have the benefit of not requiring immunosuppression. Oculopharnygeal muscular dystrophy is characterized by late onset eyelid drooping (ptosis) and dysphagia (difficulty swallowing) as a result of dystrophy of the pharyngeal muscle. Myoblasts from unaffected limb muscles were grafted into the pharyngeal muscle of patients, following on from promising preclinical trials conducted in the beagle dog 44. The trial is a safety and efficacy trial, with results on any swallowing improvements expected in 2015. A similar trial using autologous myoblasts from non-affected areas is underway for facioscapulohumeral muscular dystrophy, which is characterized by asymmetric muscle weakness, predominantly in the face, scapula and upper arms. The results of this trial are expected soon. However, it would not be possible to treat a muscular dystrophy such as DMD with autologous myoblasts because the regenerated myofibres would still lack dystrophin and therefore be prone to continuing bouts of degeneration and regeneration. Because the use of autologous cells may not require immunosuppression of the patient, efforts have been made to genetically modify autologous myoblasts. Vectors such as retroviruses and lentiviruses have been used to heritably insert marker or therapeutic, genes into myoblasts; however, retroviruses can only infect dividing cells, and so the quiescent, more ‘stem-cell’ myoblasts would not be transduced. Lentiviral vectors efficiently infect quiescent cells, including stem cells 45, and, because they integrate into the host genome, give long-term, heritable, gene expression. The drawbacks of lentiviral vectors include possible gene silencing, or mutagenesis 46, as a result of the site at which the virus inserts into the host genome. Although lentiviruses integrate preferentially into active transcription sites 47, the development of third-generation lentiviruses with an advanced self-inactivating design, to reduce transactivation of neighbouring genes 48, physiological promoters (such as muscle creatine kinase or desmin) 49, cell-specific envelope proteins 50 and enhancer-less regulatory elements (e.g. the ubiquitously acting chromatin opening element) 49,51, should reduce the risk of insertional mutagenesis or gene silencing.

A major disadvantage of lentiviruses is that they can carry only a relatively small DNA insert of up to 10 kb 52. Lentiviral vectors have been used to insert either a mini- or micro-dystrophin gene, or constructs designed to skip mutated dystrophin exons, into myoblasts 45,53,54. These genetically-modified myoblasts contribute to regenerated muscle fibres, containing a shorter dystrophin protein, after their intramuscular transplantation in animal models of DMD. Although these engineered mini-dystrophins appear to retain most of the functional properties of full-length dystrophin, they nevertheless miss important domains, such as the nitric oxide synthase-anchoring domain 55, and so an important goal is to insert as large as possible functional dystrophin construct into a lentiviral vector.

## Improving myoblast culture conditions

Why is it that myoblasts do not perform as well as satellite cells? When placed in tissue culture, the majority of satellite cells proliferate rapidly, although a minority divide slowly and it is the latter that contribute more extensively to muscle regeneration *in vivo* 56,57. Selecting for a subpopulation of quiescent myoblasts may improve their engraftment potential. Small, nongranular mouse satellite cells 39 contribute more effectively to muscle regeneration than larger, granular satellite cells from the same preparations 58, and sorting on the basis of satellite cell size and/or specific cell surface markers 59 may be able to enrich for the ‘stem’ satellite cells with enhanced muscle regenerative capacity.

The ability to guide the behaviour and fate of stem cells in culture is hindered by a limited understanding of the niche composition and the regulation that it imposes on satellite cell fate. The niche comprises both biochemical (e.g. growth factors, cytokines, receptor ligands) and biophysical (matrix stiffness, topography, fluidity, temperature, oxygen, pH) factors that direct stem cell fate 60. Modifying any of these factors can have a pronounced impact on muscle regeneration and satellite cell self-renewal.

It is now well recognized that oxygen tension is an important component of stem cell niches. Most tissue culture is performed using atmospheric levels of oxygen (20%) when, in reality, tissue levels are much lower, usually 2–9% (14.4–64.8 mmHg) depending on the tissue; even within a tissue, there is considerable variability depending on the proximity of cells to blood vessels 61–64. The neural stem cell niche has an oxygen tension in the range < 1–8% oxygen, the hematopoietic stem cell niche in the range 1–6% oxygen and the mesenchymal stem cell niche in the range 2–8% oxygen 64, whereas the thymus, kidney medulla and bone marrow can exist at 1% oxygen 63. Low oxygen levels are often referred to hypoxic when, in reality, they are normoxic for the tissue or cell in question. Culture in 20% oxygen can be toxic to cells causing DNA damage and apoptosis 61, whereas low levels of oxygen have been shown to prevent oxidative stress caused by aerobic metabolism, in turn preventing the generation of reactive oxygen species that may cause DNA damage 61,64. However, for myoblast cultures, oxygen levels are routinely uncontrolled 61,65.

Two recent studies highlight the benefits of using more physiological levels of oxygen in the cultivation of myoblasts. Duguez *et al*. 65 confirmed that atmospheric oxygen is hyperoxic for myoblasts and represses their proliferation, compared to myoblasts cultured in 5% oxygen, and suggested that the mechanism by which this occurs is via increased mitochondrial activation in hyperoxic conditions. We have also observed a decreased proliferation of satellite cell-derived myoblasts at 20% oxygen compared to 5% oxygen (D. Briggs, L. Boldrin and J.E. Morgan, unpublished results). Another study by Liu *et al*. 66 demonstrated that reducing the oxygen level further (to 1%) increases myoblast quiescence, reduces differentiation and promotes self-renewal. It was elegantly shown that hypoxia upregulates Pax7 through downregulation of miR-1 and miR206, whose expression, in turn, is controlled by the Notch signalling pathway 66. Furthermore, hypoxia conditioning was shown to enhance the efficiency of myoblast transplantation and self-renewal *in vivo* in cardiotoxin injured mdx mouse muscles 66.

**Figure 1:**
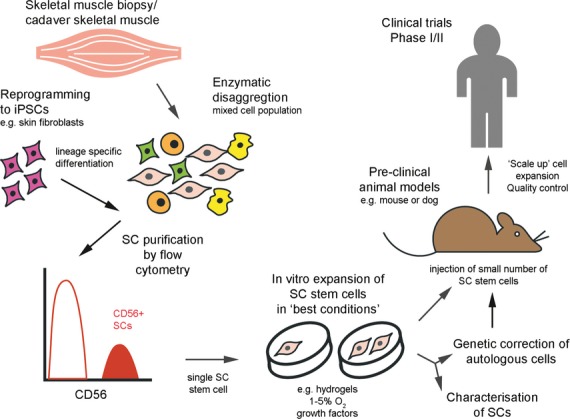
Potential protocol for improving cell therapy for muscular dystrophy. With advancements in the isolation and culture of muscle stem cells, the following may become possible. Skeletal muscle satellite cells (SCs) could be obtained by muscle biopsy or from cadaver muscle and enzymatically disaggregated to a single cell suspension containing an impure population of satellite cells. Satellite stem cells could be purified by flow cytometry. Alternatively, satellite cells could be derived from reprogrammed iPSCs. Culture conditions that allow the expansion of only the stem cell subpopulation of satellite cells would improve transplantation and require only limited cell numbers (e.g. the use of hydrogels and low levels of oxygen). Genetic correction of autologous satellite cells would also be required. Preclinical studies in animal models, such as the dystrophin deficient mdx mouse and golden retriever muscular dystrophy dog, would be performed to confirm safety and efficacy before the therapy enters the clinic. Currently, satellite cells are only deliverable intramuscularly, although further understanding of their biology may allow their modification so that they can be delivered systemically.

The importance of physiological tissue rigidity has long been suspected but, as a result of the complexity of reflecting this *in vitro*, has largely been ignored. Using a bioengineering approach, Gilbert *et al*. 67 created polyethylene glycol hydrogels, which mimic the elasticity of muscle much more closely than standard, rigid, tissue culture plastic. It was demonstrated that soft substrates enhance satellite cell survival, prevent differentiation and promote stemness (increased self-renewal) *in vitro* and, more importantly, result in extensive muscle regeneration *in vivo* compared to traditional culture on plastic 67. This was the first study to show high levels of engraftment in mice from a small number of transplanted cultured cells (100% incidence of donor-derived engraftment was obtained from 1000 cells and 10% from just 10 cells), which represents an efficiency comparable to the injection of 10 freshly-isolated satellite cells 67. The use of this artificial niche will allow the influence that other biochemical niche components have on stem cell fate and behaviour to be examined at a single cell level, on a large scale, using time-lapse microscopy and an algorithm that enables automated analysis, garnering previously unobtainable information 68. Eventually, this should allow the selection and subsequent expansion of the stem cell subpopulation of satellite cells (Fig. 1). Transplantation of satellite stem cells rather than myoblasts would dramatically improve donor-derived muscle regeneration.

Most satellite cell research is carried out using mouse cells because only very low numbers of human satellite cells can be obtained by muscle biopsy, which are then cultured to increase the cell number and thus become myoblasts. Recently, Latil *et al*. 69 showed that satellite stem cells are enriched in post-mortem tissue, adopting a dormant state and remaining viable for up to 17 days in humans and 14 days in mice. Obtaining satellite cells from post-mortem muscles could provide a large number of human normal and dystrophic satellite cells for research at the single cell level and potentially could provide autologous satellite cells for transplantation.

## Modifying the environment

**Figure 2:**
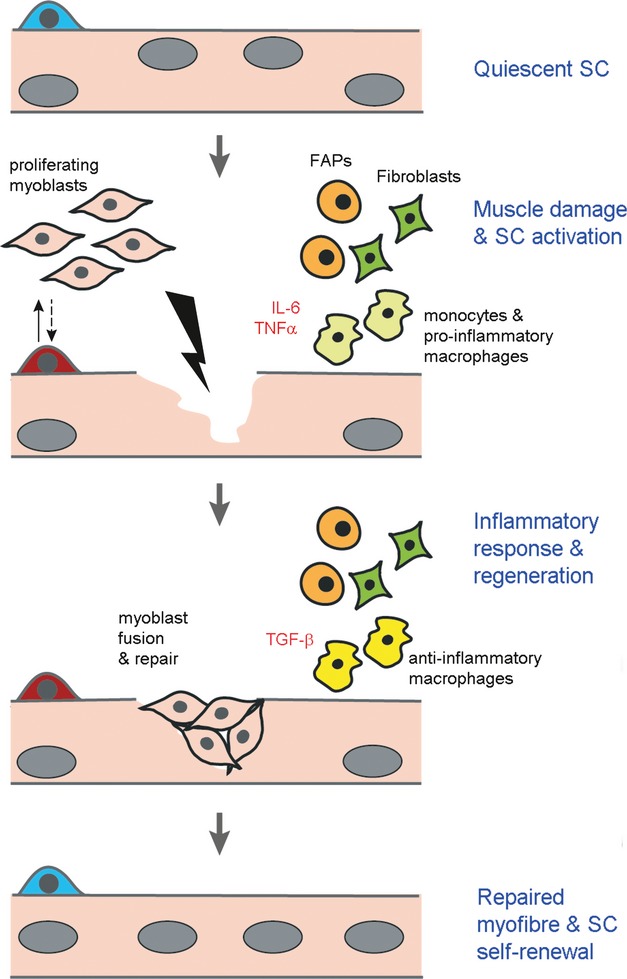
Schematic of satellite cell-mediated muscle regeneration. In response to myofibre damage, satellite cells rapidly activate and proliferate to produce a pool of myoblasts that fuse to repair or replace damaged fibres. Infiltration by immune cells occurs, including neutrophils, monocytes, pro-inflammatory and later anti-inflammatory macrophages, along with stromal cells including fibroblasts and FAPs secrete paracrine and autocrine factors, remove debris and ensure efficient regeneration. The immune and stromal cells do this by controlling the balance between myoblast proliferation and differentiation and ensuring satellite cell self-renewal to replenish the stem cell niche. A hallmark of regenerated fibres in the mouse is the central (i.e. opposed to peripheral) position of nuclei. IL-6, interleukin-6; TNFα, tumour necrosis factor α.

Satellite cells are absolutely necessary for muscle regeneration 24–26,70; however, they do not work alone (Fig. 2). Regeneration is a multistep process requiring resident and infiltrating immune and stromal cells to remove debris, regulate satellite cell proliferation and differentiation, and allow muscle remodelling 71,72. The necessity of the inflammatory response has been demonstrated in many studies, with a reduced entry of monocytes/macrophages into injured muscle causing a delay in regeneration and the persistence of adipocytes 71–74. Moreover, complete depletion strikingly results in no regenerative response, highlighting the importance of inflammation 72–75.

Coinjection of pro-inflammatory (but not anti-inflammatory) macrophages, along with human myoblasts, into regenerating muscle (injured by cryodamage) of Rag2^−/−^γC^−/−^ immunodeficient mice improves donor-derived muscle regeneration by extending the window of proliferation, increasing migration and delaying differentiation 76. It is suggested that pro-inflammatory macrophages can then switch to an anti-inflammatory phenotype *in vivo* to stimulate differentiation of the donor myoblasts 76. These results provide the first *in vivo* evidence for pro-inflammatory macrophages having a supportive role in the regulation of myoblast behaviour after engraftment into pre-injured muscle 76. A similar study, using the coinjection of mouse macrophages and myoblasts, but into the dystrophic environment of mdx mice, also reported improved donor-derived regeneration, which was attributed to improved donor myoblast survival, proliferation and migration 77. The increased survival was considered to be a result of macrophages improving cell adhesion, thereby decreasing ankiosis and having a mitogenic effect by secreting growth factors. This is important in the context of cell therapy because massive early cell death, poor proliferation and migration are some of the main obstacles that need to be overcome for it to become a viable therapy option 77.

Another vital component of the regenerating niche is muscle connective tissue (MCT) cells (stromal cells), including fibroblasts and dual potential fibro/adipoprogenitors (FAPs) 78. Fibroblasts are necessary for extracellular matrix and collagen synthesis and an increase in extracellular matrix is a hallmark of regenerating muscle. The study of MCT fibroblasts had been limited by the lack of specific markers until the recent finding that MCT fibroblasts express the transcription factor Tcf4 79. Using genetic ablation studies, Murphy *et al*. 25 showed that Tcf4+ fibroblasts are required for efficient regeneration, and that their loss leads to premature satellite cell differentiation, depletion of the myoblast pool and smaller regenerated fibres. Reciprocally, myoblasts promote MCT fibroblast proliferation 25. FAPs have only recently been described but represent a significant fraction of the mononuclear cells present in muscle 80. FAPs are quiescent in healthy muscle but proliferate efficiently in response to damage; their transient expansion during regeneration provides signals that promote the terminal differentiation of proliferating myoblasts 80. A greater understanding of this population of cells may lead to therapeutic strategies for reducing the scarring and fibrosis found in dystrophic muscle, thereby providing an environment amenable to muscle regeneration 80.

The effect of ageing on satellite cell function is a matter of much debate because the loss of skeletal muscle mass and function with increasing age (sarcopenia) is of great importance in ageing western populations. However, despite evidence that the satellite cell niche deteriorates with age 81 and that satellite cells are lost with age 58,82, the regeneration-competent, ‘stem’ satellite cells are retained and those derived from aged donors remain as functional as those from young donors 9,58,83,84. It therefore appears that there are two subpopulations of satellite cell: one that is lost with age and is responsible for maintaining muscle mass, and a second that is retained throughout life 9 and, given the correct environmental cues, can contribute robustly to muscle regeneration.

## Improving regeneration

There is a plethora of studies in mice examining ways of augmenting the regenerative potential of myoblasts. Preventing cell death, increasing proliferation and/or migration, and decreasing early differentiation have all been shown to have a positive impact on mouse and human myoblast transplantations in immunodeficient mice. For example, upregulating the heatshock response (Hsp70 protein) improves both mouse and human myoblast survival, leading to increased engraftment 85,86. Reducing hypoxia-related death by overexpressing vascular endothelial growth factor has a similar effect 87. Overexpression of matrix metalloproteinase 9, a proteolytic enzyme that can remodel the extracellular matrix, enhances myoblast migration and engraftment 88. Transforming growth factor-β (TGF-β) is a negative regulator of skeletal muscle development and elevated levels can limit skeletal muscle regeneration 89. Fakhfakh *et al*. 90 have shown that treatment with oral losartan, a molecule that downregulates TGF-β1 expression, improves the transplantation efficiency of human myoblasts into immunodeficient dystrophic mice, as demostrated by an increase in dystrophin positive fibres 1 month after engraftment compared to nontreated controls 90. Increased myoblast survival was observed 3 days after transplantation (10% versus 6% of radiolabelled cells), which led to increased proliferation and differentiation concomitant with the increased expression of Myf5, MyoD and myogenin 90. Blocking the myostatin signal (another negative regulator of muscle regeneration) with a dominant negative receptor improves the success of human myoblast transplantation by increasing myoblast proliferation and fusion and changing the expression of myogenic regulatory factors 91. However, this approach may not be as straightforward as hoped; a recent clinical trial using ACE-031 (a soluble form of activin receptor type IIB, which binds to myostatin and other members of the TGFβ family) in DMD patients, was terminated early because of safety concerns (http://www.acceleronpharma.com/products/ace-031/; ClinicalTrials.gov Identifier: NCT01099761).

Concerning the limited migratory capacity of human myoblasts *in vivo*, several studies have linked this with the limited proliferation of the injected myoblasts. When human myoblasts are injected into the cryodamaged muscles of Rag2^−/−^γC^−/−^ immunodeficient mice, in medium containing serum, which is rich in growth factors, rather than NaCl/P_i_, the window of proliferation is extended from 3 to 5 days. This increases migration, leading to enhanced regeneration as a direct result of slower myoblast differentiation 92. Similarly, AG490 (a specific inhibitor of janus tyrosine kinase 2) has been used to block myoblast differentiation, increasing proliferation and cell survival *in vivo* 93. However, other studies have shown that, although co-injection of insulin-like growth factor 1 and/or basic fibroblast growth factor with human myoblasts improves myoblast migratory capacity and dispersal 94,95, growth factor addition does not improve the transplantation success in undamaged primate muscle 95, in contrast to the enhanced regeneration observed in mice 92,96.

## Challenges remaining

### How to induce regeneration

A major problem with the intramuscular injection of myoblasts in the human clinical trials was that regeneration (dystrophin positive fibres) appeared to be limited to damaged muscle along the injection trajectory. This was also seen in primate experiments 40,95. In mice, successful engraftments require either pre-treatment of the host muscle with irradiation 97,98, or an injury to be administered to induce or increase muscle damage; with use of the snake venom myotoxins notexin and cardiotoxin 99 or cryodamage 100 being most common. Irradiation limits the host satellite-cell contribution to regeneration and provides an optimal environment for donor mouse cell engraftment 84,98,101. Cryodamage destroys cells near to the injury site but preserves the basal lamina of muscle fibres 102. Following cryodamage, skeletal muscle can regenerate, indicating that at least some satellite cells either survive the injury or migrate into damaged areas 84. Injection of myotoxins destroys muscle fibres but preserves their basal lamina, nerves, blood vessels and satellite cells 84. Neither cryodamage, nor myotoxins are as effective as irradiation for enhancing mouse donor satellite cell-derived muscle regeneration 84. This is not the case for human myoblasts, where cryodamage is at least as effective as irradiation, allowing similar amounts of donor muscle regeneration and engraftment of more total donor cells (including cells outside of muscle fibres) 96,103. The reason for differences between the behaviour of mouse and human myoblasts is not known, suggesting caution with respect to the assumption that what works in mice will also work in humans. For patients in whom it would be unethical to use these pre-treatments, other ways of increasing donor satellite cell or myoblast engraftment might exist. Intense muscle exercise has been shown to greatly improve myofibre regeneration in mdx mice 104. It is possible that exercise (rather than an acute and extensive injury to the host muscle) may be sufficient to promote donor-derived muscle regeneration in patients.

### Harnessing the potential of induced pluripotent stem cells (iPSCs)

iPSCs 105 hold great promise for cell therapy; they could potentially yield unlimited numbers of autologous stem/progenitor cells. This is important because myoblasts, particularly dystrophic ones, undergo a limited numbers of doublings before entering senescence and the use of donor heterologous myoblasts requires life-long immunosuppression. A caveat is that patient-derived iPSCs would still need to be genetically corrected before transplantation. The generation and use of human iPSCs does not pose the same ethical dilemma as deriving human embryonic stem cells (ESCs), making them a more attractive candidate 106,107. The technology for both ESCs and iPSCs is limited by the efficiency of cell-lineage-specific differentiation and the efficiency of cell purification to eliminate the risk of teratoma, although many strategies are being devised to overcome these limitations 108. Reprogramming of mouse iPSCs and ESCs into satellite cells/myoblasts has been achieved using various protocols 109–110, although the equivalent reprogramming of human iPSCs and ESCs has lagged behind. Only one reported study, showing reprogramming of human ESCs into myoblasts with limited efficiency 112, was available until Darabi *et al*. 106, Tedesco *et al*. 113 and Goudenege *et al*. 107 published new protocols for deriving myogenic progenitors from iPSCs, based on mesoderm commitment followed by myogenic transcription factor overexpression, within a few months of each other. Darabi *et al*. 106 and Goudenege *et al*. 107 demonstrated very efficient reprogramming of both human iPSCs and ESCs using the forced overexpression of different myogenic regulatory factors, specifically MyoD in an adenoviral vector 106 and Pax7 in a lentiviral vector 107. Both methods gave highly efficient myogenic conversion, with cells expressing high levels of the satellite cell marker CD56 and myosin heavy chain upon *in vitro* differentiation, and notably generating a large number of muscle fibres upon intramuscular transplantation into immunodeficient dystrophic mice 106,107 Darabi *et al*. 106 also demonstrated a functional improvement in treated muscles, long-term expression of donor-derived dystrophin (11 months) and occupation of the satellite cell niche. Tedesco *et al*. 113 used a similar strategy but went one step further by deriving mesoangioblast-like cells (no CD56 expression) from human iPSCs generated from limb-girdle muscular dystrophy 2D (sub-type of limb-girdle muscular dystrophy) patient fibroblasts or myoblasts. These cells were then lentivirally transduced with both a therapeutic gene (*Sgca*, encoding α-sarcoglycan) to correct the genetic defect and with MyoD to induce myogenic differentiation. Importantly, donor cell engraftment into *Sgca*-null immunodeficent mice, was obtainable using both intramuscular and inter-arterial injections, as indicated by α-sarcoglycan expression 113. However, there are safety concerns with iPSCs, particularly the potential tumourigenicity of cells that are not fully differentiated at the time of transplantation, as well as the genomic integrity of the iPSCs 114.

## Concluding remarks

In recent years, there has been both an improved understanding of the biology of satellite cells themselves, together with increasing knowledge on the effect of the host skeletal muscle environment on their function *in vivo*. In particular, the effect of factors such as microRNAs, growth factors and extracellular matrix components produced by host cells, including myofibres, blood vessel-associated, stromal and inflammatory cells, and the effect of the host satellite cell niche on donor satellite cell engraftment are particularly relevant to improving donor cell engraftment. We envisage that a combination of tissue culture conditions to promote or retain the ‘stem-like’ myoblasts, with modification of the host muscle environment to enhance donor satellite cell migration, proliferation and self-renewal, will be the way forward.

Because satellite cells and their progeny myoblasts 15 do not appear to be systemically deliverable, they could only be used to treat individual muscles, although this might still be of benefit to patients with DMD. If hand or finger muscles could be successfully treated, this could improve the quality of life 115 by allowing the patient, for example, to operate a computer keyboard or touchscreen.

Even in the era of molecular therapies, myoblast or other stem cell therapies are still highly relevant. Although potential treatments for DMD such as exon skipping are promising, exon skipping is neither applicable to all DMD patients, nor will it restore lost muscle fibres. An effective stem cell-based treatment will therefore be a powerful alternative, or adjunct, to other therapies.

## Abbreviations

DMD: Duchenne muscular dystrophy
ESC: embryonic stem cell
FAP: fibro/adipoprogenitor
iPSC: induced pluripotent stem cell
MCT: muscle connective tissue
SC: satellite cell
TGF-β: transforming growth factor-β
